# Near-Infrared Forearm Vascular Width Calculation Using Radius Estimation of Tangent Circle

**DOI:** 10.3390/bioengineering11080801

**Published:** 2024-08-07

**Authors:** Qianru Ji, Haoting Liu, Zhen Tian, Song Wang, Qing Li, Dewei Yi

**Affiliations:** 1 Beijing Engineering Research Center of Industrial Spectrum Imaging, School of Automation and Electrical Engineering, University of Science and Technology Beijing, Beijing 100083, China; 42023067@xs.ustb.edu.cn (Q.J.); m202211458@xs.ustb.edu.cn (Z.T.); liqing@ies.ustb.edu.cn (Q.L.); 2 Department of Nephrology, Peking University Third Hospital, Beijing 100191, China; 3 Department of Computing Science, University of Aberdeen, Aberdeen AB24 3UE, UK; dewei.yi@abdn.ac.uk

**Keywords:** near-infrared image, vascular width, image enhancement, skeleton extraction, edge detection, geometric characteristics, APSLSE image segmentation, radius estimation of tangent circle (RETC)

## Abstract

In response to the analysis of the functional status of forearm blood vessels, this paper fully considers the orientation of the vascular skeleton and the geometric characteristics of blood vessels and proposes a blood vessel width calculation algorithm based on the radius estimation of the tangent circle (RETC) in forearm near-infrared images. First, the initial infrared image obtained by the infrared camera is preprocessed by image cropping, contrast stretching, denoising, enhancement, and initial segmentation. Second, the Zhang–Suen refinement algorithm is used to extract the vascular skeleton. Third, the Canny edge detection method is used to perform vascular edge detection. Finally, a RETC algorithm is developed to calculate the vessel width. This paper evaluates the accuracy of the proposed RETC algorithm, and experimental results show that the mean absolute error between the vessel width obtained by our algorithm and the reference vessel width is as low as 0.36, with a variance of only 0.10, which can be significantly reduced compared to traditional calculation measurements.

## 1. Introduction

At present, relevant research results show that chronic kidney disease (CKD) affects 15% to 20% of adults worldwide and increases the risk of cardiovascular disease outcomes, which is one of the most common diseases worldwide [1,2]. CKD is estimated to become the fifth leading cause of death globally by 2040 [3]. The gradual decline of the kidney leads to the accumulation of uremic solutes, with a negative effect on organs, and particularly on the cardiovascular system [4]. The progression of CKD to the end stage requires renal replacement therapy, with hemodialysis accounting for approximately 298.4 people per million people worldwide [5], making it the main method of renal replacement therapy [6]. The establishment and maintenance of a well-functioning permanent vascular pathway is a necessary condition to ensure long-term dialysis for patients, which will directly affect their prognosis [7,8,9]. Hemodialysis requires the establishment of vascular access, and the dialysis vascular access, known as the “lifeline”, often faces related complications such as stenosis, thrombosis, pseudoaneurysm, swollen hands [10,11], etc. Therefore, monitoring the functional status of blood vessels has become crucial.

**Figure 1:**
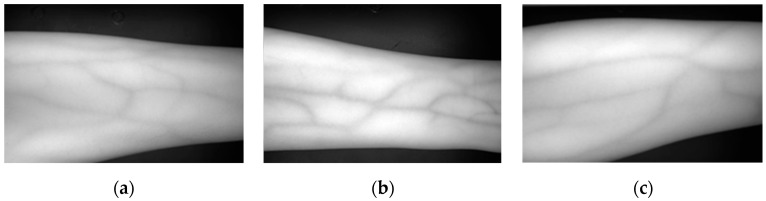
Near-infrared forearm venous angiography of different subjects. (**a**) Male with low body fat. (**b**) Female with high body fat. (**c**) Male with high body fat.

Ultrasound and near-infrared images have been widely used in medical settings in recent years. Ultrasound imaging has emerged as a promising non-invasive, real-time, valid, and reliable tool [12]. Ultrasound imaging has proven to be highly versatile, swiftly scanning soft tissues, and excelling in soft tissue imaging [13]. However, the vascular images collected by ultrasound have the problem of insufficient clarity, and the cost of ultrasound equipment is also high, making it inconvenient to use. Unlike ultrasound imaging, near-infrared images have the characteristics of light invariance and detail preservation [14,15], making near-infrared images clearer. In addition, near-infrared devices have low cost and are easy to operate. As shown in Figure 1, the near-infrared images of forearm venous vessels from different subjects are presented. Considering the advantages of near-infrared imaging in vascular structure imaging, we chose near-infrared vascular imaging as the research object. By observing and analyzing near-infrared vascular images, we hope to more accurately evaluate vascular function and disease status, providing a more reliable reference for clinical diagnosis and treatment.

This paper proposes a blood vessel width calculation algorithm for forearm near-infrared venous vessel images based on the radius estimation of the tangent circle (RETC). This method first preprocesses the initial image through the steps of cropping, denoising, two-stage image enhancement, and image segmentation [16,17]. Then, the Zhang–Suen refinement algorithm [18] is used to extract the vascular skeleton. The Zhang–Suen refinement algorithm mainly achieves skeleton extraction through two-stage iteration. Next, the Canny edge detection algorithm [19] is used to extract the edges of blood vessels. Finally, combining geometric knowledge, we develop a RETC algorithm to calculate the vessel width, fit the vessel width with the diameter of the tangent circle, and output the vessel width value. Our work makes the following contributions to the field: (1) we propose a RETC algorithm, which effectively calculates the vessel width in near-infrared images of the forearm, providing a new method for vascular width measurement. (2) We combine several image-processing techniques, including image preprocessing, Zhang–Suen skeleton extraction, Canny edge detection, and our RETC approach. This integration forms a comprehensive and efficient system for calculating blood vessel width in near-infrared images, enhancing the accuracy and reliability of measurements. (3) The development of this algorithm and system provides more reliable and effective tools and methods for vascular health monitoring and disease management and has important clinical application potential.

In the following sections, the proposed computational flow chart is presented in Section 3, the developed calculation algorithm is illustrated in Section 4, and some experiments and discussions are shown in Section 5. A conclusion is made in Section 6.

## 2. Related Works

### 2.1. Image Enhancement

In the field of medical image analysis, common image enhancement methods include histogram equalization [20], the nonlinear unsharp mask algorithm [21], the frequency domain transformation method [22], the directional filtering method [23], etc. The authors in Ref. [24] combined histogram equalization with wavelet transform to achieve image denoising. In Ref. [25], an adaptive anti-sharpening mask algorithm was proposed based on region segmentation, which had a good processing effect on edge enhancement. The authors of Ref. [26] applied wavelet transform to vascular images, mainly studying the enhancement processing methods for their low-frequency parts. The above three image enhancement methods, although theoretically effective, are likely to result in the loss of some vascular information in practical applications due to the lack of consideration for the directionality of blood vessels. In Ref. [27], a vascular image enhancement method using a directional adjustable filter was proposed. This method used directional adjustable filters to extract venous vessels in various directions, employed wavelet transform for image fusion, and finally utilized an improved nonlinear anti-sharpening mask algorithm to obtain contrasting and prominent vascular images. In Ref. [28], a histogram and blur algorithms were combined to enhance images. Based on Gabor filtering and Retinex theory, Ref. [29] incorporated a fuzzy algorithm to effectively enhance finger vein images. In Ref. [30], directional filter banks and Hessian filters were used to achieve better results in vascular imaging processing. However, the objects processed by the above two methods are high-quality vascular images with high contrast and low noise. In the actual venous imaging process, the quality of infrared venous vascular images is often poor due to various environmental factors. Therefore, it is difficult to provide reliable and accurate venous information in practical applications.

The current image enhancement methods have good results in processing high-quality and high-contrast images, but in practical applications, due to insufficient consideration of vascular directionality, environmental noise, and contrast issues, it is often difficult to provide reliable and accurate vascular information. This paper uses a two-stage image enhancement method to enhance images from both local and global perspectives, which can better handle the details of vascular images.

### 2.2. Calculation of Vascular Width

Common methods for calculating vascular width include model-based methods [31], clustering algorithm-based methods [32], mathematical fitting-based methods, and graph theory-based methods [33]. Based on graph theories, Ref. [34] first transformed the dual boundary segmentation problem of retinal blood vessels into an issue of two 3D surfaces, and then calculated the thickness of blood vessels by transforming it into a problem of calculating the minimum closed set in a node-weighted graph. In Ref. [35], the authors used the Sobel operator to detect the edges of retinal blood vessels and performed cubic spline fitting. In Ref. [36], a method for measuring the diameter of blood vessels in fundus images based on prior knowledge was proposed, which utilized the spatial position and directional information of the vascular skeleton and an improved directional local contrast method to detect the boundaries of blood vessels from smooth images. Unlike the approach of calculating vessel thickness by detecting vessel width mentioned above, Ref. [37] fitted a parameter model of retinal vessel image intensity distribution to obtain an estimated value of vessel thickness. For the venous vessels on the back of the hand, Ref. [38] used a Gaussian model of venous vessels to measure the thickness of venous vessels on the back of the hand. In Ref. [39], the authors extracted the centerline and skeleton of coronary arteries based on the Hessian matrix and realized the measurement of coronary artery diameters in digital subtraction angiography (DSA) images. In Ref. [40], a blood vessel diameter measurement method based on the clustering algorithm was proposed, and Ref. [41] focused on the width estimation of coronary arteries.

Although many methods for calculating blood vessel width have been proposed, there are still problems in practical applications. One of the issues is that the width of blood vessels in vascular images may vary with changes in position, and traditional width calculation methods cannot fully consider local vascular changes, resulting in inaccurate calculation results. Therefore, the calculation method of blood vessel width in forearm near-infrared images still needs further research.

## 3. Proposed Computational Method

**Figure 2:**
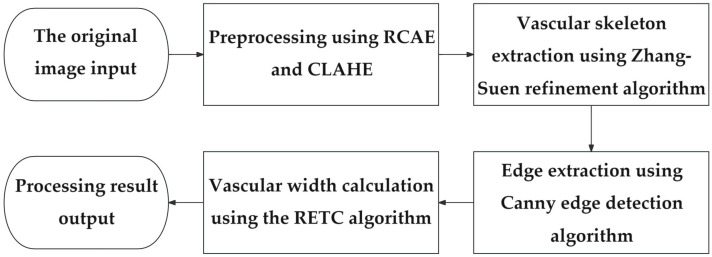
Algorithm flowchart of near-infrared blood vessel width computation. The processes of the proposed algorithm include image preprocessing, skeleton extraction, edge detection, and vascular width calculation.

This paper focuses on the issues of blurred near-infrared forearm blood vessel segmentation and width estimation. The traditional method may be affected by factors such as irregular, blurred, or interrupted blood vessel edges; our method utilizes the geometric features and morphological information to accurately detect and extract blood vessel edges. In Figure 2, the proposed computation steps are as follows. First, image preprocessing is performed. Due to the fact that the processed image is captured by an infrared camera, there may be issues with blurred blood vessel boundaries, excessive noise, and uneven brightness in the initial image. Therefore, cropping, filtering, enhancement, and segmentation are performed. The enhancement first performed is residual convolutional auto-encoder (RCAE) enhancement [42], followed by contrast-limited adaptive histogram equalization (CLAHE) [43]. Second, we obtain the pixel point coordinates of the blood vessel skeleton. This step uses the Zhang–Suen refinement algorithm to extract the skeleton [44]: the first stage removes endpoint pixels from the image, and the second stage removes fork pixels from the image. These stages are iteratively applied until no pixels can be deleted. Third, the Canny edge extraction method is used to obtain vascular boundaries. Canny edge detection includes Gaussian smoothing, gradient amplitude and direction calculation, non-maximum suppression, and dual-threshold edge tracking. Finally, considering the direction and edge features of the vascular skeleton, we develop a RETC algorithm to calculate the blood vessel width. The average diameter of the tangent circle obtained at the skeleton point is considered as the width value of the blood vessel.

## 4. Proposed Vascular Width Calculation Method

### 4.1. Image Preprocessing

**Figure 3:**
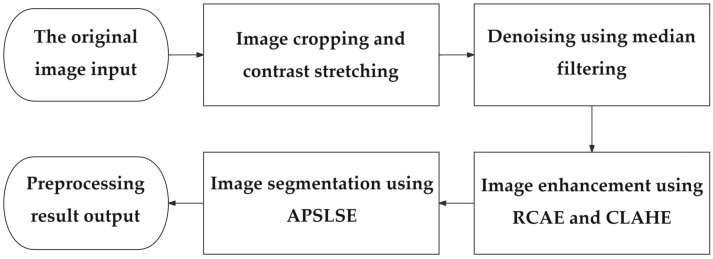
Preprocessing computational flowchart.

The near-infrared images of superficial blood vessels in the forearm are easily affected by near-infrared acquisition equipment and various experimental factors, which often have characteristics such as blurriness, high noise, and low contrast between the vascular area and the surrounding tissue area [45,46]. In order to obtain clearer vascular structure images, the preprocessing performed in this paper includes image cropping and background removal, contrast stretching and noise removal, image enhancement, and image segmentation. The preprocessing process is shown in Figure 3. First, the image is cropped and the background is removed to eliminate interference from non-arm regions. Due to the influence of ambient light and near-infrared camera aperture, the grayscale of the target area after removing the background is relatively concentrated. Contrast stretching can effectively increase the gap between vascular areas and other tissue areas such as skin, bones, and muscles, making subsequent segmentation easier. Noise removal can effectively reduce the noise introduced during image acquisition, and using median filtering for denoising can solve the problem of blurred image details.

Regarding the image enhancement, this paper uses a two-stage image enhancement method, which first performs RCAE image enhancement and then CLAHE image enhancement. RCAE enhancement can preserve the local details of the image, while CLAHE enhancement can improve the overall contrast. The two-stage image enhancement method combines the advantages of convolutional neural networks and traditional image enhancement methods, which can effectively strengthen vascular structures and suppress other interference. RCAE is constructed based on an unsupervised learning convolutional auto-encoder (CAE), which extracts effective features from input data by using convolution and deconvolution operations [47,48]. RCAE processes images by recursively applying convolutional kernels multiple times, gradually enhancing the contrast and clarity of images. Each recursive convolution operation takes into account the local neighborhood information of pixels in the image to adapt to the characteristics of the image. The CLAHE algorithm evolved from the histogram equalization (HE) algorithm, which improves image quality by adjusting contrast limits [49]. It divides the image into many small blocks, equalizes the histogram of each block, and introduces contrast constraints during equalization to avoid excessive noise enhancement. Then, interpolation is used to eliminate boundary artifacts, making the enhanced image smoother.

Image segmentation uses a type of adaptive prior shape level set evolution (APSLSE) method [50]. The APSLSE model utilizes rough segmentation results as initial contour iterations, effectively reducing the impact of initial contours on segmentation performance. APSLSE uses the length of the zero-level curve or surface of level and function *ϕ* to obtain the zero-level set contour. The length of the zero-level curve or surface of level set function *ϕ* is represented by Formula (1).

(1) $$L_{g}\left(\phi \right)≜\int_{\Omega }g^{*}\delta \left(\phi \right)|\nabla \phi |dx$$

where *L_g_*(*ϕ*) is the length term of the energy function, *Ω* is the entire image domain, g* is the edge indicator function, *δ* is the Dirac function, and *∇* is the gradient operator.

In practical applications, the Dirac function *δ* is approximated by the smoothing function (2) for *L_g_*(*ϕ*).

(2) $$\delta_{2,\varepsilon }\left(x\right)=\left\{\begin{array}{cc} \frac{1}{2\varepsilon }[1+\cos\;(\frac{\pi x}{\varepsilon })],\;\;\; & |x|\leq \varepsilon \\ 0, & |x|>\varepsilon \end{array}\right.$$

where *ε* is a parameter and *ε* = 2 in this paper; the definition of edge indicator function g* is shown in (3).

(3) $$g^{*}≜\frac{1}{1+{\left|{\nabla G}_{\sigma }*I\right|}^{2}}$$

where *G_σ_* is a Gaussian kernel with a standard deviation of *σ*; *I* is the image to be processed. Clearly, APSLSE segmentation introduces flexible prior shapes to make the active contour more adaptable to the shape and boundary changes of the target, thereby improving the accuracy of segmentation.

### 4.2. Extraction of the Vascular Skeleton

In this paper, extracting the vascular skeleton is to obtain the centerline of blood vessels, which facilitates the subsequent acquisition of vascular width information. The main requirements for extracting vascular skeletons are to ensure the connectivity of blood vessels and preserve the original vascular trends and other detailed features. The skeleton should be a single pixel and the computational speed should be fast to match the extraction targets.

**Figure 4:**
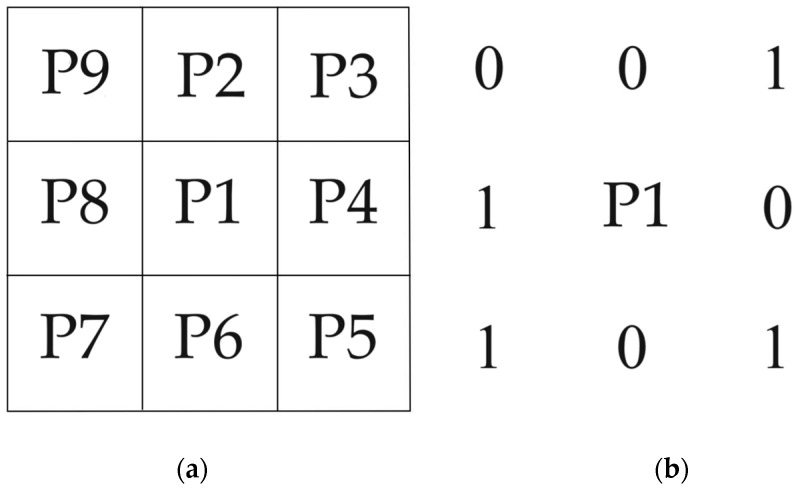
Pixel position map. (**a**) Diagram of the eight neighboring pixels around the center pixel P1. (**b**) Diagram example of P1 and its eight neighbors.

This paper uses the Zhang–Suen refinement algorithm to extract vascular skeletons. The pseudocode of Zhang-Suen refinement algorithm is given in Appendix A. The refinement algorithm can meet the above requirements very well. It can extract the main structure and morphological features of blood vessels in an image, remove redundant information, and help analyze and understand the shape and structure of blood vessels. Compared to other refinement algorithms, the Zhang–Suen refinement algorithm has simpler calculations and better refinement effects; it can refine the object boundaries in binary images into a pixel-wide skeleton to preserve the structural features of the object. Assuming the image is a binary image with a black background and a value of 0, the foreground blood vessels to be refined are white with a value of 1. As shown in Figure 4a, a 3 × 3 area in an image is labeled with names P1, P2, P3, …, and P9 for each point, where P1 is located at the center of the image and P1 = 1 is a white pixel. The main steps of the Zhang–Suen refinement algorithm are as follows:

1. The first round of iteration: for each pixel P1, the algorithm checks the surrounding eight pixels. The pixel P1 that simultaneously meets the following four conditions will be marked as pending deletion.
  (1) The number of white pixels in adjacent pixels around P1 ranges from two to six.
  (2) The number of changes in adjacent pixels P2, P3, P4, P5, P6, P7, P8, and P9 around P1 shall not exceed one.
  (3) The number of white pixels in adjacent pixels P2, P4, and P6 around P1 should not be less than one.
  (4) The number of white pixels in adjacent pixels P4, P6, and P8 around P1 should not be less than one.
2. For each foreground pixel P1, the algorithm checks the surrounding eight pixels again. The pixel P1 that simultaneously meets the following four conditions are marked for deletion.
  (1) The number of white pixels in adjacent pixels around P1 ranges from two to six.
  (2) The number of changes in adjacent pixels P2, P3, P4, P5, P6, P7, P8, and P9 around P1 shall not exceed one.
  (3) The number of white pixels in adjacent pixels P2, P4, and P8 around P1 should not be less than one.
  (4) The number of white pixels in adjacent pixels P2, P6, and P8 around P1 should not be less than one.
3. After the first and second iterations, the algorithm removes all foreground pixels marked as pending deletion from the image.
4. The algorithm checks whether the image has changed after the deletion operation. If the image has not changed, it indicates that the refinement has been completed and the algorithm can be terminated; otherwise, the algorithm returns to the first iteration and proceeds to the next iteration.

Taking Figure 4b as an example, in the first iteration process, if the second condition is not met, the adjacent pixels P2, P3, P4, P5, P6, P7, P8, and P9 around P1 change three times, exceeding once. Due to the fact that only pixels that meet all four conditions can be marked as pending deletion, in the example in Figure 4b, P1 cannot be marked as pending deletion.

### 4.3. Vascular Edge Detection

The purpose of edge detection is to significantly reduce the data size of an image while preserving its original image attributes. Edge detection of vascular images can provide edge feature information for subsequent acquisition of vascular width. The main requirements for vascular edge extraction in this paper are strong noise resistance, accurate edges, sufficiently fine edge images, and fast running speed. After comparisons, the gradient-based edge extraction method determines the edge position by calculating the gradient amplitude and direction of pixels, which is usually effective and can meet the above requirements. Common gradient algorithms include the Canny operator, Sobel operator [51], Prewitt operator [52], Roberts operator [53], etc. The numerical calculation methods of Sobel, Prewitt and Roberts operators are given in Appendix B. In this paper, the Canny edge detection algorithm is utilized. The Canny operator not only effectively suppresses noise but also has high detection accuracy [54]. The brief steps of the Canny algorithm for edge detection are as follows:

1. Denoising: to reduce the impact of noise, Gaussian filtering is first applied to the image. The Gaussian filtering can blur images, making noise more evenly distributed in the image. The formula for a Gaussian filter is shown in Formula (4), where *G*(*x*, *y*) is the output of the Gaussian filter, *x* and *y* are the spatial coordinates of the filter, and *η* is the standard deviation of the Gaussian kernel.
  (4) $$G\left(x,y\right)=\frac{1}{2\pi \eta^{2}}e^{-(x^{2}+y^{2})/2\eta^{2}}$$
2. Gradient estimation: Canny uses Sobel and other operators to calculate gradient amplitude and direction on the smoothed image. The gradient direction can help determine the direction of edges. The calculation formulas for gradient amplitude *GM* and gradient direction *GD* are shown in Formulas (5) and (6), respectively, where *G_x_* is the gradient of the image in the *x* direction and *G_y_* is the gradient in the *y* direction.
  (5) $$GM=\sqrt{{G_{x}}^{2}+{G_{y}}^{2}}$$
  (6) $$GD=\arctan\;(\frac{G_{y}}{G_{x}})$$
3. Non-maximum suppression: a type of suppression is applied to the gradient map, filtering out non-edge pixels and making blurry boundaries clearer. This process preserves the local maximum values in the gradient direction of each pixel and filters out other values.
4. Dual threshold detection: both a high threshold and a low threshold are considered in Canny. If the gradient amplitude of a pixel is greater than the high threshold, it is marked as a strong edge. On the other hand, if the gradient amplitude is between the low and high thresholds, it is marked as a weak edge. Otherwise, it will be marked as a non-edge.
5. Edge tracking: based on the connectivity of strong edges, the weak edges connected to them are marked as edges, while other weak edges are deleted.

### 4.4. The RETC Algorithm

Vascular width is an important indicator in the medical diagnosis process. Abnormal or drastic changes in vascular width can be characterized as symptoms [55]. In this paper, the basic principle of the RETC algorithm is as follows. After extracting the blood vessel skeleton and edges from a near-infrared image, a circle is drawn with each pixel on the blood vessel skeleton as a center, and the radius of the circle is gradually increased until the first intersection point is formed between the circle and the blood vessel boundary. At this point, the radius growth of the circle is stopped. The diameter of the circle is the pixel value of the blood vessel diameter at the skeleton point. Finally, we take the average pixel value of the blood vessel diameter obtained at each vascular skeleton point as the final width value of venous vessel segmentation. The pseudocode of the RETC algorithm is given in Appendix C.

**Figure 5:**
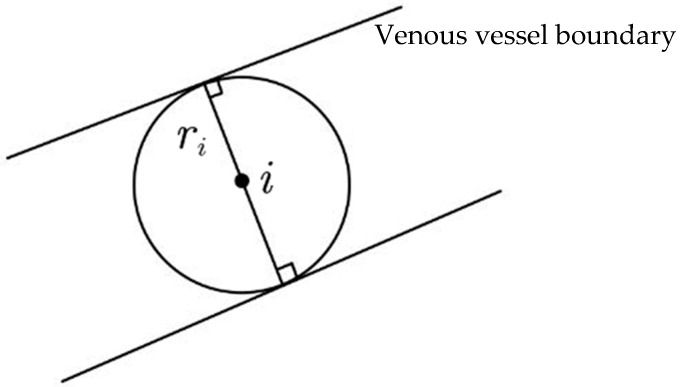
Venous vascular diagram.

Figure 5 is a schematic diagram of a venous vessel, where the venous skeleton has only one unit pixel width and a total length of *n* pixels. Taking the *i* skeleton point as an example, we can regard the *i* skeleton point as the center of the circle and create a gradually increasing radius circle until it intersects with the blood vessel boundary. At this point, the number of pixels occupied by the diameter of the circle is equal to the vein thickness *d_i_* at the *i* skeleton point.

If the radius of the circle at the *i* skeleton point is denoted as *r_i_*, then the vein width *d_i_* at the *i* skeleton point can be calculated using Formula (7).

(7) $$d_{i}=2r_{i}-1$$

From this, the average width *D* of the venous vessel segmentation can be obtained, and this can be used as an estimation of the width of sub-vein segmentation. The calculation formula for the width *D* of the venous vessel in this segmentation is shown in Formula (8).

(8) $$D=\frac{1}{n}\sum_{i=1}^{n}d_{i}$$

At the algorithm implementation level, the RETC algorithm mainly uses a cyclic structure to traverse the vascular skeleton points. In the outer loop, we create a circle with a gradually increasing radius centered on each vascular skeleton point. In the inner loop, the distance between the vascular skeleton point and each edge point is calculated, and a conditional judgment statement is used to determine whether it is less than or equal to the current radius. If any edge points intersect with the circle of the current radius, it is considered that the corresponding blood vessel edge has been found. If we find the edge point that intersects with the circle, we record the radius of the current circle and jump out of the loop. This is because only one intersection point needs to be found to determine the edge of the blood vessel. Finally, we record all radii found at intersection points and store them in a list, which can be used to calculate the diameter or other features of blood vessels.

## 5. Experiments and Discussion

### 5.1. Experiment System and Data

**Figure 6:**
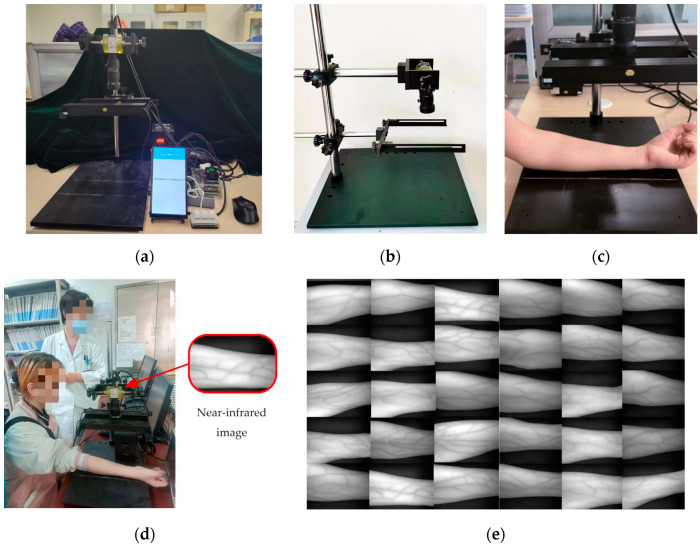
Experiment equipment and data. (**a**) The entire collection device. (**b**) Near-infrared camera and forearm placement table. (**c**) Application case of the proposed device. (**d**) Photo example of subject experiment. (**e**) Image data samples.

This paper establishes a near-infrared image acquisition system for collecting a dataset of near-infrared images of superficial blood vessels in the forearm. A near-infrared image acquisition system is designed based on the principle that hemoglobin in the blood has a significantly different ability to absorb and reflect near-infrared light compared to other tissues around blood vessels. This system uses two bar-shaped near-infrared light sources to illuminate the surface of the forearm, while using a near-infrared camera for photography. At this point, hemoglobin in the blood absorbs more near-infrared light, resulting in a darker color, while other physiological tissues such as skin and muscles absorb less near-infrared light, resulting in a lighter color. In addition, in order to reduce the impact of uneven lighting during image acquisition, two strip light sources are placed parallel and fixed at a height of about 8–10 cm from the surface of the forearm. Through experimental comparison, it can be found that among near-infrared light sources with wavelengths of 660 nm, 850 nm, and 940 nm, near-infrared light with a wavelength of 850 nm has the best effect in obtaining images of superficial blood vessels in the forearm. Meanwhile, in order to avoid interference from other wavelengths of light, a narrow-band filter of 850 nm ± 10 nm is installed at the lens. A basic near-infrared image dataset of superficial blood vessels in the forearm can then be established using the near-infrared image acquisition system for further research. Finally, our dataset consists of two batches of 742 images, mainly from young students aged 18 to 28, with a male-to-female ratio of approximately 3:1. During the training and testing stages, the data are processed into a grayscale image of 100 × 100 pixels and simulated using Python 3.7 programming on a PC (i5-1035G1 CPU1.00 GHz, 16 GB RAM, and NVIDIA GeForce MX 350). The near-infrared forearm vascular image acquisition device and the collected partial data are shown in Figure 6.

### 5.2. Evaluation of Image Enhancement Algorithm

**Figure 7:**
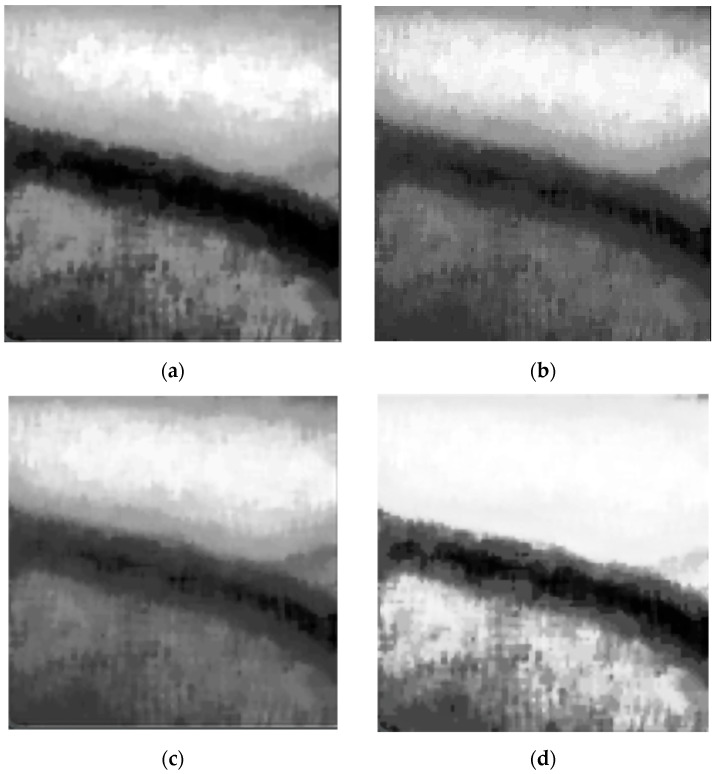
Experimental results of image enhancement algorithms. (**a**) The results of HE. (**b**) The results of AHE. (**c**) The results of SSR. (**d**) The results of RCAE and CLAHE.

In order to evaluate the effectiveness of the image enhancement algorithm, this section compares four algorithms, including RCAE and CLAHE two-stage enhancement algorithms, the HE algorithm, the adaptive histogram equalization (AHE) [56] algorithm, and the single-scale Retinex (SSR) [57] algorithm. The four algorithms are applied to the same pre-cropped and median-filtered vascular image, and the experimental results are shown in Figure 7.

The HE algorithm expands the dynamic range of an image by reallocating the grayscale of its pixels and makes the histogram of the image more uniform. This can enhance the contrast of the image, making the details in the image clearer and more visible. The HE algorithm is a global enhancement method that strips the grayscale distribution of the entire image to improve the overall contrast, but it can lead to problems such as excessive enhancement or contrast disturbance. As shown in Figure 7a, the HE algorithm has a significant enhancement effect on the vascular area, but the distinction between the background area and the vascular area is not clear enough.

AHE enhances the contrast of an image by applying histogram equalization to local regions of the image. Unlike global histogram equalization, AHE divides the image into many small regions and performs histogram equalization within each small region. This can better handle local contrast changes in the image and preserve the image details. However, it can lead to inconsistent local contrast, making the image appear less smooth or natural. As shown in Figure 7b, the AHE algorithm’s enhanced detailing of the vascular structure cannot be clearly displayed, resulting in poor performance.

The SSR algorithm is based on Retinex theory, which reduces local contrast changes in the image while maintaining global contrast. Its principle is based on the separation of illumination and reflection, achieved by decomposing and reconstructing images in the logarithmic domain. It can effectively balance the global and local contrast of an image, making it appear more natural and smoother. However, when processing images with complex textures, the SSR algorithm cushions them from noise amplification, which can easily lead to a decrease in image quality. As shown in Figure 7c, the SSR algorithm is also unable to clearly display the details of the vascular structure, and the contrast between the vascular area and the background area is not high enough.

Finally, the corresponding results show that the two-stage image enhancement method of RCAE and CLAHE can effectively handle contrast distortion and noise amplification while maintaining image details, with lower computational complexity, achieving the best enhancement effects among all the enhancement algorithms in this section.

### 5.3. Evaluation of Vascular Skeleton Extraction Algorithm

**Figure 8:**
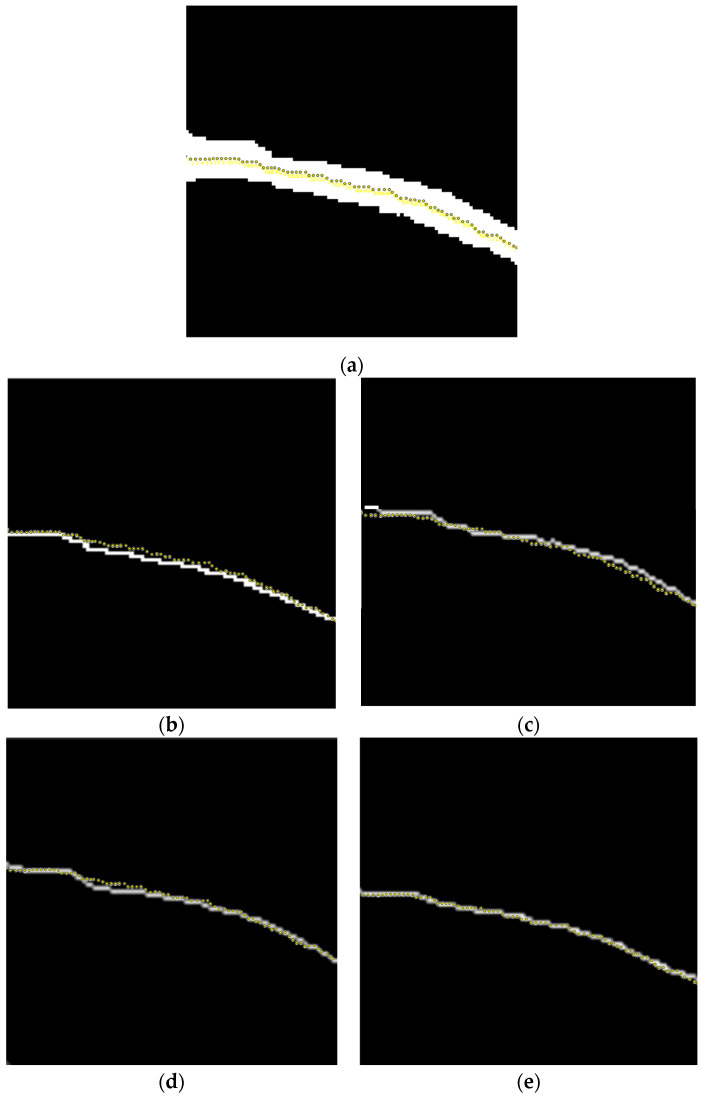
Experimental results of different vascular skeleton extraction algorithms. (**a**) Hand-labeled vascular skeleton. (**b**) The result of the morphological refinement algorithm based on Hit and Miss. (**c**) The result of the skeleton extraction algorithm based on judgment templates. (**d**) The result of the Hilditch refinement algorithm. (**e**) The result of the Zhang–Suen refinement algorithm.

In order to compare the performance of different vascular skeleton extraction algorithms, the Zhang–Suen refinement algorithm, a morphological refinement algorithm based on Hit and Miss [58], a skeleton extraction algorithm based on judgment templates [59], and the Hilditch refinement algorithm [60] are used on the same forearm near-infrared vascular image for performance evaluation. In addition, this paper manually marks the vascular skeleton on the preprocessed vascular image, providing a ground truth, and overlays it with the vascular skeletons obtained by different algorithms for clearer comparison to determine the accuracy of the algorithm. The results are shown in Figure 8.

The morphological refinement algorithm based on Hit and Miss uses structural elements and calculates the matching situation between image elements and structural elements during the iteration process. Then, based on the matching situation, a corrosion operation is performed, and the iteration is carried out until there are no further changes. From Figure 8b, it can be seen that the algorithm obtains many inflection points from the vascular skeleton, which are not smooth enough. This is because the algorithm adopts a simple corrosion operation, so it cannot handle the overlapping between structural elements, which leads to some inaccurate refinement effects. In addition, it can be seen from Figure 8b that there is a particularly large number of spiky structures on the vascular skeleton obtained by the algorithm. The shape of blood vessels in vascular images is usually very irregular, and the Hit and Miss algorithm is prone to generating unnecessary details and spikes in the skeleton extraction process when processing these irregular shapes.

The skeleton extraction algorithm based on judgment templates updates the central pixel according to the situation of surrounding pixels to achieve the effect of refinement. The performance of this algorithm highly depends on well-designed judgment templates, and designing suitable templates may require some experience and domain knowledge. This paper uses a 16 × 16 binary judgment template to adapt to the shape and characteristics of the vascular skeleton. From Figure 8c, it can be seen that there is a significant deviation between the position of the vascular skeleton obtained by this algorithm and the manually marked position of the vascular skeleton in Figure 8a. The vascular skeleton obtained by this algorithm has significant inflection points in some areas, and the refinement results generated by this algorithm are not smooth enough, with jagged edges that affect the clarity of the vascular structure. For small and complex structures, the refinement effect of a judgment template-based algorithm is not as good as the Zhang–Suen refinement algorithm, which is prone to losing important detail information.

The basic idea of the Hilditch refinement algorithm is to iteratively use a set of refinement templates to remove edge points from the image until there are no more points that can be refined. The Hilditch refinement algorithm is simple to implement, easy to understand, and can maintain the topological structure of objects. However, in the processing of some complex structures, the refinement results of this algorithm are not as stable as the Zhang–Suen algorithm, and there may be discontinuous or incomplete situations. As shown in Figure 8d, using the manually labeled vascular skeleton as a reference, the algorithm obtains an imprecise vascular skeleton. The extracted vascular skeleton has a large inflection point on the left side, which leads to insufficient smoothness and excessive refinement, and the effect is not as good as the Zhang–Suen refinement algorithm.

After comparison, it can be found that the Zhang–Suen refinement algorithm can generate smooth and accurate vascular skeletons, effectively preserving the overall structure and morphology of blood vessels, and it can match well with the ground truth. Refining the result to a single-pixel-width skeleton is beneficial for subsequent image analysis and processing. When dealing with complex vascular images, the Zhang–Suen refinement algorithm exhibits strong robustness and can maintain the coherence and completeness of the refinement results.

### 5.4. Evaluation of Vascular Edge Detection Algorithm

**Figure 9:**
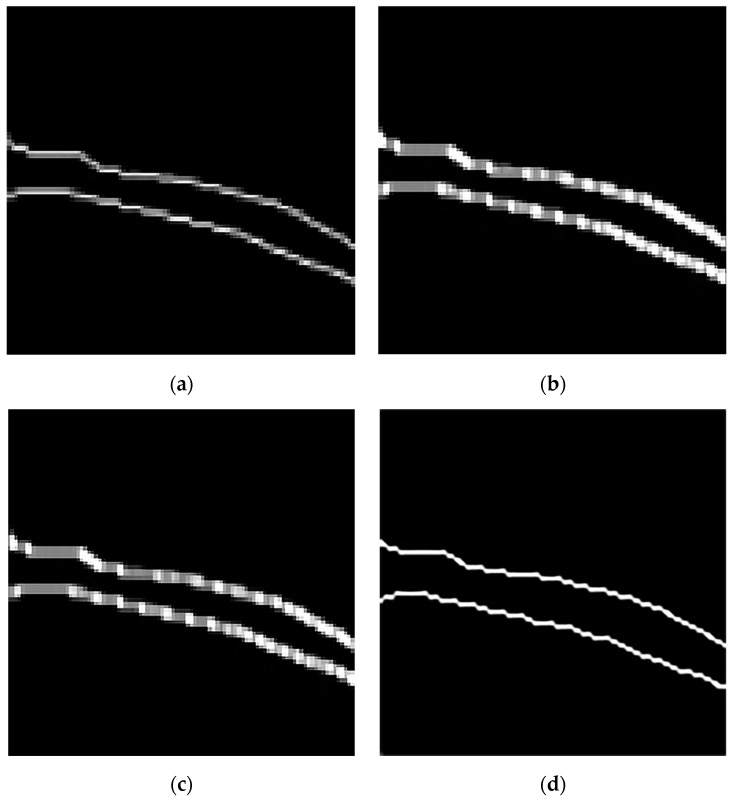
Experiment results of different blood vessel edge detection methods. (**a**) Result of the Roberts operator. (**b**) Result of the Sobel operator. (**c**) Result of the Prewitt operator. (**d**) Result of the Canny operator.

In order to compare the performance of blood vessel edge detection algorithms, four operators, namely the Canny operator, the Roberts operator, the Sobel operator, and the Prewitt operator, are applied to the same near-infrared forearm vein vessel image to evaluate their processing effectiveness. The results are shown in Figure 9.

The Roberts operator is based on a pair of small convolution kernels, each corresponding to edge detection in the horizontal and vertical directions. These two convolution kernels calculate the difference in grayscale values between pixels to detect edges in the image. In this way, the Roberts operator can find edges in the image and identify their directions. This operator performs well on images with steep edges and low noises, especially those with more positive and negative 45-degree edges, but its positioning accuracy is poor. As shown in Figure 9a, the results obtained by this algorithm lose some edges and do not have the ability to suppress noise.

The Sobel operator applies two convolution kernels, i.e., horizontal and vertical, to the image to calculate the gradient amplitude of each pixel in the horizontal and vertical directions. Then, it determines the position of the edge based on the gradient amplitude. The calculation process of the Sobel operator is relatively simple and the calculation speed is fast. Meanwhile, due to the use of local averaging by the Sobel operator, it can effectively avoid the influence of noise. However, as shown in Figure 9b, the Sobel operator performs edge extraction on the binarized image of forearm veins, resulting in thicker edges and multi-pixel widths.

Prewitt utilizes the difference generated by pixel grayscale values within a specific area to achieve edge detection. Due to the Prewitt operator using a 3 × 3 template to calculate pixel values within a region, while the Robert operator has a 2 × 2 template, the edge detection results of the Prewitt operator are more pronounced in both horizontal and vertical directions than the Robert operator. The Prewitt operator has a certain inhibitory effect on noise, but it does not consider the impact of distance between adjacent points on the current pixel. As shown in Figure 9c, the edge localization accuracy obtained by this algorithm is relatively low, and there is also the problem of multi-pixel width.

From the experimental results, it can be seen that the edge detection extracted by Canny is clear, smooth, and has a single pixel width, resulting in the best effect. The Canny operator can perform non-maximum suppression and dual-threshold detection during edge extraction, effectively suppressing noise and improving edge connectivity and accuracy, making it excellent and more reliable for extracting edges from vascular images.

### 5.5. Evaluation of RETC Algorithm

This section evaluates the computational effectiveness of the RETC algorithm. We compare the circular structure-based method [61], graph theory-based method [34], cubic spline fitting-based method [35], decision tree-based method [37], multi-scale matching filtering-based method [62], Block Matching and 3D Filtering (BM3D) and multi-scale line detection method [63], and Width Attention-based convolutional neural Network (WA-Net) method [64] with the geometric prior model-based method proposed in this paper. According to classical statistical theory [65,66], the minimum number of statistically significant experiments for evaluation should be larger than 25. The above six methods are applied to 40 near-infrared forearm vein vessel images and evaluate the estimated vessel width values.

This paper evaluates different methods using the mean and variance of the absolute value *e_i_* of the difference between the estimated blood vessel width ${\bar{w}}_{i}$ of the *i* image and the reference blood vessel width *w*. The relationship between the difference *e_i_* between ${\bar{w}}_{i}$ and *w* is shown in Formula (9). Formulas (10) and (11), respectively, provide the calculation formulas for mean *μ_e_* and variance *δ_e_*, where *n* represents the use of *n* images in the experiment. In this experiment, *n* = 40.

(9) $$e_{i}=|{\bar{w}}_{i}-w|$$

(10) $$\mu_{e}=\frac{1}{n}\sum_{i=1}^{n}e_{i}$$

(11) $$\delta_{e}=\frac{1}{n}\sum_{i=1}^{n}{\left(e_{i}-\mu_{e}\right)}^{2}$$

**Figure 10:**
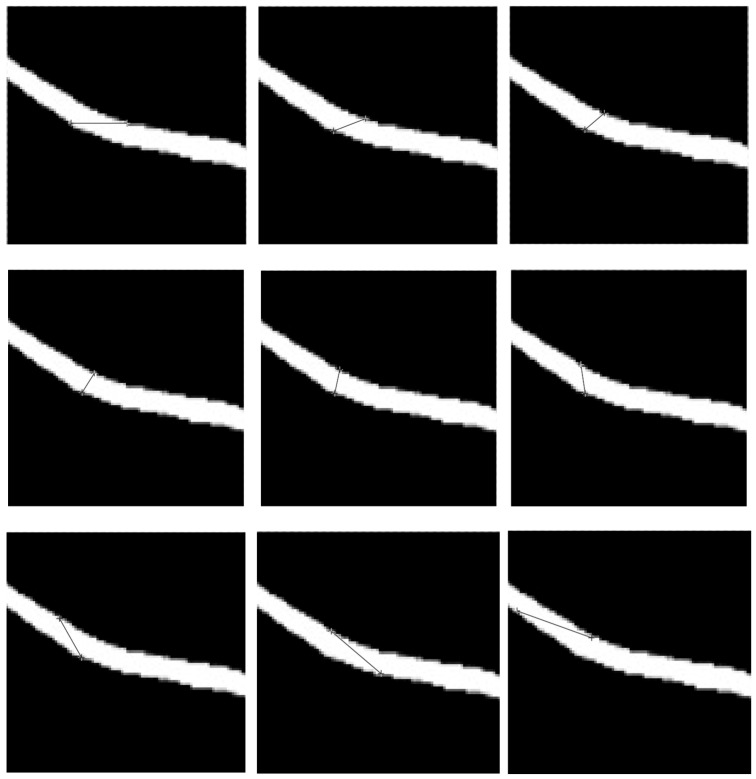
Measurement method for reference vessel width. The black lines in figures represent the ruler tool.

The measurement method for reference vessel width is as follows: in the near-infrared image of forearm venous vessels, we measure the width of vessels manually. We arbitrarily select a position of the blood vessel, place the ruler horizontally on top of the blood vessel, and read the number of blood vessel pixels displayed by a ruler tool, denoted as N. Then, with a step size of 20°, we rotate the ruler and record one N value for each rotation until the angle between the ruler and the image is 160°. A total of nine values of N are recorded. Then, we select the smallest of these nine values as the width of the blood vessel. The measurement process is shown in Figure 10: from left to right, and from top to bottom, the ruler is measured from 0° to 160°, with N values of 23.0, 13.9, 10.6, 9.4, 10.2, 12.2, 18.4, 26.5, and 31.9, respectively. Therefore, the minimum value is taken, and the width of the blood vessel is 9.4 pixels.

Table 1 presents the comparison results between our method and several existing methods for calculating blood vessel width.

The circular structure-based method firstly segments the blood vessels and then measures them on each sub-segment. On the basis of obtaining the center point of the blood vessel, this method calculates the width of the sub-segment arteries and veins through the radius and angle of the circle, and then estimates the width of the entire blood vessel by averaging the sub-segment vessel width. As shown in Table 1, although the circular structure-based method has good performance, the *μ_e_* and *δ_e_* obtained are larger than those of the methods proposed in this paper. Therefore, the accuracy of the circular structure-based method for calculating the vessel width is not as good as that of the method proposed in this paper.

The graph theory-based method for calculating vascular width uses the image’s vascular skeleton to create a graphical structure. It analyzes paths and connections within this structure to determine vascular width. Initially, the method extracts the vascular skeleton from the image using morphological operations. Then, it employs a path analysis algorithm from graph theory to calculate path length and connections, indirectly inferring blood vessel width. The effectiveness of this method highly depends on the quality of vascular skeleton extraction. If the extraction of the vascular skeleton is inaccurate or incomplete, it will lead to inaccuracies in the final calculation of vascular width. As shown in Table 1, the *μ_e_* and *δ_e_* obtained by this method are both high, indicating that the calculation effect of blood vessel width is average.

The method of calculating blood vessel width based on cubic spline fitting uses cubic spline curves to fit the edges of blood vessels, and then calculates the width of blood vessels by analyzing the characteristics of spline curves. On the basis of obtaining the edges of blood vessels, this method uses cubic spline curves to fit the edge points of blood vessels, in order to obtain a smooth blood vessel boundary curve. Then, this method selects an appropriate position on the fitted vascular boundary curve and measures the width of the curve. The parameter selection during the spline fitting process has a significant impact on the final result, requiring repeated adjustment and optimization, which increases the complexity and time consumption of this algorithm. As shown in Table 1, the *μ_e_* obtained by this method is very high, indicating that the accuracy of the algorithm is relatively low.

The blood vessel width calculation method based on a decision tree utilizes a decision tree algorithm to classify pixels in blood vessel images and obtain the width of blood vessels. For each vascular pixel, it first extracts some features as input to the decision tree, and then uses the labeled vascular image dataset to construct a classifier using the decision tree learning algorithm. During the training process, the decision tree classifies vascular pixels based on feature values to distinguish between vascular and non-vascular pixels. Finally, it infers the width of blood vessels based on the classification results. Decision trees are prone to overfitting the training data during the training process, especially when the data volume is small or the feature dimension is high, which can lead to insufficient generalization ability in this model. As shown in Table 1, the *μ_e_* of this method is very high, indicating low accuracy and poor performance.

The multi-scale matching filtering-based method utilizes multi-scale matching filters to filter vascular images and enhance their edge and texture features. Then, it detects the edge contours of blood vessels and uses the filter responses of different scales to estimate the width of blood vessels. Finally, based on the filter response and the distribution of edge contours, the width of blood vessels is calculated. This method involves the selection of multiple parameters, such as the scale and shape of filters, and the selection of parameters has a significant impact on the results. Moreover, this method is sensitive to noises in the image, which can affect the response of filters and the results of edge detection, thereby affecting the accuracy of calculating blood vessel width. As shown in Table 1, the *μ_e_* and *δ_e_* obtained by this method are relatively high, indicating insufficient accuracy and stability.

BM3D is an advanced image-denoising technology. This technology first identifies and groups similar 2D image blocks into a 3D array, then shrinks the transformation domain of the 3D array to effectively remove noise while preserving image details. Finally, the filtered 3D blocks are transformed back to their original positions and aggregated to form the denoised image. Multi-scale line detection is used to identify and analyze vascular structures at different scales. This method applies algorithms designed specifically for enhancing tubular structures, such as Frangi filters, which have a strong response to linear structures, thus achieving effective blood vessel detection. The width of blood vessels is measured by analyzing the response of line detection filters at different scales. However, BM3D requires a large amount of computation, which is not suitable for real-time applications due to block matching and 3D collaborative filtering steps. Additionally, line detection methods may lead to blurred blood vessel edges, affecting the accuracy of width estimation. As shown in Table 1, the *μ_e_* of this method is relatively high.

WA-Net is a deep learning model that combines width and attention mechanisms. First, this method standardizes the input vascular images and increases the diversity of training data through data augmentation techniques. Then, multiple convolutional layers are used to extract low-level features from the vascular image, including edge, texture, and shape information. Finally, the extracted features are input into the regression layer to directly predict the width of blood vessels. Due to the use of multi-layer convolutional networks and attention mechanisms, WA-Net requires a large amount of computational resources for both training and inference processes, making it unsuitable for real-time applications. The performance of deep learning models heavily relies on large-scale, high-quality annotated data. For vascular width estimation, a large number of manually annotated vascular image datasets are required, which may be difficult to obtain in practical operations. As shown in Table 1, the *δ_e_* of this method is relatively high.

According to the experimental results in Table 1, it can be seen that the *μ_e_* and *δ_e_* obtained by our method are smaller than those of other methods, indicating that our RETC vascular width calculation algorithm has the best accuracy and stability.

**Figure 11:**
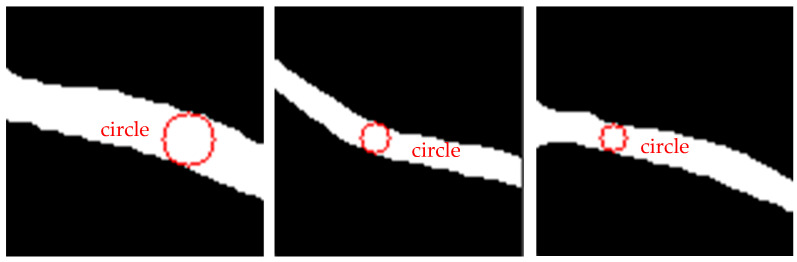
Inscribed circle image samples. The red graphs represent the inscribed circles that intersect with the edges of blood vessels obtained using the RETC algorithm.

Figure 11 shows the inscribed circle image samples of different blood vessel images processed using the method proposed in this paper. It can be seen that the inscribed circles obtained by our method can fit well with the edges of the blood vessels. Therefore, the blood vessel width value obtained from this inscribed circle is also relatively accurate.

### 5.6. Discussion

Near-infrared imaging technology has broad application prospects in the field of medical imaging, and this paper aims to promote the development of near-infrared blood vessel analysis technologies. Vascular width is an important indicator reflecting the health status of blood vessels. Studying the calculation method of near-infrared vascular width can provide doctors with objective evaluation indicators of vascular health status and assist clinical diagnosis and treatment decision-making. Due to the non-invasive and non-radioactive characteristics of near-infrared vascular imaging technology, it can evaluate human blood vessels without damaging the skin. Therefore, this paper contributes to the development of a safe and non-invasive vascular assessment method, providing technical support for early diagnosis and monitoring of diseases such as kidney disease. Meanwhile, applying this paper to the puncture operation during hemodialysis can improve the accuracy and success rate, reduce the incidence of complications, and thus improve the treatment effect and quality of life of patients.

In order to analyze the functional status of forearm blood vessels, this paper proposes the RETC algorithm. The RETC algorithm analyzes and processes images of near-infrared forearm blood vessels, achieving measurement of blood vessel width without the need for traumatic surgery or injection, avoiding additional harm to patients and meeting the non-invasive needs of clinical and scientific research. This algorithm can quantitatively analyze the width of blood vessels and provide specific blood vessel diameter data, rather than just qualitatively describing the condition of blood vessels. This quantitative analysis ability helps doctors and researchers to more accurately evaluate the severity of vascular lesions and diseases, providing strong support for clinical diagnosis and scientific research. Compared to manual measurement of vascular width, the RETC algorithm adopts an automated image-processing algorithm, which can quickly analyze and measure a large number of vascular images, improve work efficiency, save time and cost, and meet the high efficiency needs of medical institutions and research teams.

In this paper, we combine the traditional CLAHE algorithm with the deep learning algorithm RCAE to achieve the best image enhancement effect. Regarding skeleton extraction and edge detection, this paper chooses to use the traditional Zhang–Suen refinement algorithm and Canny edge detection technique, respectively. This is because new machine learning algorithms typically require a large amount of annotated data for training. However, in many medical image-processing tasks, collecting annotated data is costly and time-consuming, especially when manual annotation by experts is required. Meanwhile, machine learning algorithms, especially deep learning models, often require high-performance computing resources and longer training times, which may not be practical for some resource-limited medical applications. The traditional algorithms used in this paper have high accuracy and low computational complexity, and can run quickly on ordinary computers, making them suitable for real-time or near-real-time application scenarios. When calculating the width of blood vessels, this paper uses the newly proposed RETC algorithm. The RETC algorithm can combine geometric knowledge to calculate the diameter of blood vessels using tangent circle fitting.

In this paper, we also comprehensively consider the prospect of using the RETC algorithm to calculate vessel width. First, the computational complexity of RETC is relatively low. The low computational complexity makes it suitable for real-time applications and integration into clinical workflows without imposing significant computational overhead. Second, from the perspective of computational effectiveness, the RETC algorithm can provide an accurate estimation of vessel width. Many experimental results show that compared with traditional width estimation algorithms, RETC exhibits higher robustness and accuracy in handling changes in vascular morphology. Regarding algorithm innovation, the RETC algorithm achieves accurate measurement of blood vessel width by introducing the concept of tangent circles, and it performs well in handling irregular and complex-shaped blood vessels. In terms of future portability and inheritance, the RETC algorithm has also shown good adaptability. In future, with the continuous advancement of computing technology, the RETC algorithm can be ported to more efficient hardware platforms, significantly accelerating the computing process and meeting the needs of real-time applications.

**Figure 12:**
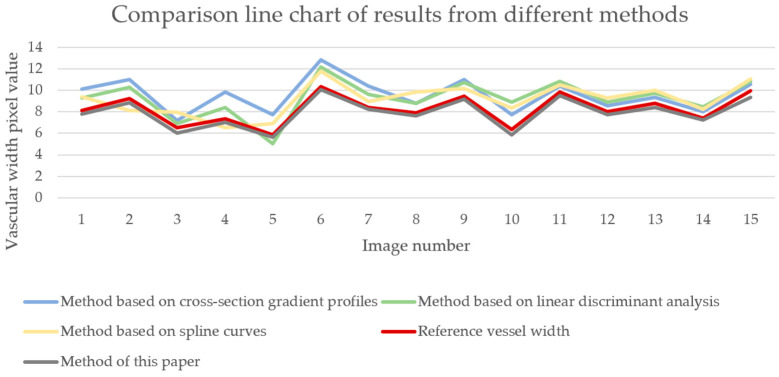
Line chart comparison of results from different blood vessel width calculation methods.

This paper also attempts other methods to obtain vascular width information. An experimental cross-sectional gradient profile-based method [67], a linear discriminant analysis (LDA)-based method [68], and a spline curve-based method [69] are analyzed. We applied the above three methods to 15 vascular images and compared the results with the manually measured baseline vascular width. As shown in Figure 12, it can be seen that the results obtained by the above three methods differ significantly from the actual blood vessel width values and have poor stability, making it difficult to accurately obtain the blood vessel width of the forearm near-infrared venous vessel image. The main reason for this result is that the method based on cross-sectional gradient contours estimates the width of blood vessels by detecting the gradient contours of blood vessel cross-sections, which requires high-quality blood vessel images. When there are situations such as unclear blood vessel contours, blurred edges, or fractures in the image, the accuracy of width estimation by this method will be significantly reduced. The LDA-based method utilizes linear discriminant analysis to distinguish between vascular and non-vascular regions, and extracts features from them to estimate vascular width. However, due to the possible overlap or small changes in grayscale and the texture features of blood vessels and surrounding tissues in near-infrared vascular images, LDA is unable to effectively distinguish between vascular and non-vascular regions, thereby affecting the accurate estimation of width. The method based on spline curves represents the contour of blood vessels by fitting spline curves, extracting vascular width information from them. However, due to the irregular contour and significant curve changes of blood vessels in near-infrared vascular images, there are fitting errors in the spline curve fitting process, resulting in inaccurate extraction of width information.

This paper also conducted other supplementary experiments in order to evaluate algorithm performance. We randomly selected 10 images from the collected near-infrared forearm vascular image dataset, of which five were left forearm vascular images of a 20-year-old male and the other five were left forearm vascular images of a 20-year-old female. Then, we used the RETC algorithm on these images separately to obtain the results of blood vessel width. Table 2 provides specific information, vascular width calculation results, and calculation accuracy for these 10 images. The average accuracy of male vascular width calculation was 0.958, and the average accuracy of female vascular width calculation was 0.954. It can be seen that the average calculation accuracy of male vascular width obtained using the RETC algorithm is slightly higher than that for females. This is because there is a difference in the diameter of blood vessels between males and females. Generally, the diameter of blood vessels in males is larger than that in females. Therefore, at the same image resolution, the measurement error of blood vessel diameter in males may be smaller.

Our results indicate that the RETC algorithm provides precise and reliable measurements, which are crucial for clinical and diagnostic purposes. The innovative aspect of the RETC algorithm lies in its novel approach to vessel width estimation, which differs from traditional methods that often rely on more complex and computationally intensive techniques. Moreover, the RETC algorithm has demonstrated high adaptability and portability, ensuring its future application in various clinical settings. Currently, the RETC algorithm has not yet been implemented in practice. However, future plans involve integrating this algorithm with a custom-developed near-infrared blood vessel image acquisition device. We have established a collaboration with Peking University Third Hospital (PUTH) to bring this technology into clinical use. We aim to capture near-infrared images of patients’ forearms at PUTH and apply the RETC algorithm to calculate and analyze blood vessel widths. This practical application will not only validate the effectiveness of our algorithm in a clinical setting but also contribute to improving patient care by providing accurate measurements that can inform medical decisions, particularly in the monitoring and maintenance of arteriovenous fistulas.

This paper has significant implications for the field of medical image processing. The RETC algorithm utilizes mathematical models and image-processing techniques to quickly and accurately calculate vascular width, making it suitable for rapid clinical screening and monitoring. The RETC algorithm can provide quantitative analysis results of vascular width, quantify vascular abnormalities and disease changes, evaluate the functional status of blood vessels, and provide an objective reference for clinical diagnosis and treatment. The RETC algorithm can be applied to various near-infrared vascular images and is suitable for measuring vascular width in different populations and parts of the body. Clearly, our method also has certain limitations. For instance, the instability of the quality of forearm near-infrared vascular images can affect the accuracy of vascular boundary extraction, which in turn affects the calculation results of vascular width. Meanwhile, the performance of the RETC algorithm also depends on the quality of input images. Variations in image quality, such as noise and low contrast, can impact the accuracy of the vascular width calculations. Moreover, the current preprocessing steps might not adequately handle all variations in image conditions, leading to inconsistencies in the results. The algorithm’s computational efficiency also requires further optimization to be practical for real-time applications in a clinical setting. Based on the limitations above, our future research can be conducted from the following aspects: we can adopt more accurate and stable boundary detection algorithms, combined with various image-processing technologies such as edge detection and segmentation by machine learning, to improve the accuracy and stability of blood vessel boundaries. Additionally, enhancing the preprocessing techniques to robustly handle a wider range of image qualities and exploring advanced machine learning methods for more reliable feature extraction and segmentation will be crucial. These improvements will help in addressing the current limitations and advancing the applicability of the RETC algorithm in diverse clinical scenarios.

## 6. Conclusions

This paper combines geometric knowledge and proposes the RETC algorithm, which completes the task of extracting vascular width information from near-infrared images of the forearm. First, image enhancement and segmentation techniques are used to preprocess the image, making key vascular locations clearer. Second, the Zhang–Suen refinement algorithm is used to obtain the vascular skeleton. Third, the Canny edge detection algorithm is used to extract the vascular edge coordinates. Finally, the RETC algorithm is used to obtain vascular width information. This study is characterized by its integration of multiple image-processing techniques to achieve high precision in vascular width extraction. The combination of enhancement, refinement, and edge detection methods ensures robust performance across varied image qualities. The proposed RETC algorithm, grounded in geometric principles, offers a novel approach to accurately determining vascular widths, setting a foundation for future advancements. In the future, further optimization and improvement is needed in this paper to enhance the accuracy, stability, and efficiency of the blood vessel width calculation algorithm, which can provide more reliable and effective tools and methods for vascular health monitoring and disease management. Future research will focus on refining these techniques to handle a wider range of imaging conditions and patient variations, as well as exploring real-time applications in clinical settings.

## Figures and Tables

**Table 1:** Comparisons of the effectiveness of different blood vessel width calculation methods.

Methods	$\mu_{e}$	$\delta_{e}$
Circular structure [61]	0.49	0.16
Graph theory [34]	1.12	0.30
Cubic spline fitting [35]	1.31	0.28
Decision Tree [37]	2.14	0.42
Multi-scale matched filtering [62]	1.05	0.57
BM3D and multi-scale line detection [63]	0.83	0.24
WA-Net [64]	0.41	0.22
Method of this paper	0.36	0.10

**Table 2:** Statistical table of image information and vascular width calculation results.

Features	1	2	3	4	5	6	7	8	9	10
Gender	male	male	male	male	male	female	female	female	female	female
Age	20	20	20	20	20	20	20	20	20	20
Height (cm)	182	163	175	165	180	156	160	160	162	172
Weight (kg)	62	62	56	60	75	44	52	65	60	56
Vascular width	10.57	8.46	8.09	9.2	9.12	7.82	8.72	9.02	9.29	8.73
Reference vascular width	11.2	8.7	8.5	9.6	9.4	8.1	9.2	9.5	9.9	9.1
accuracy	0.94	0.97	0.95	0.96	0.97	0.97	0.95	0.95	0.94	0.96

## Data Availability

The data presented in this study are available on request from the corresponding author, Haoting Liu.

## Appendix A. Pseudocode for the Zhang–Suen Refinement Algorithm

The Zhang–Suen thinning algorithm is a widely used algorithm for skeletonizing binary images. This algorithm iteratively removes pixels from the boundaries of objects while preserving the connectivity of the image, resulting in a skeleton that is one pixel wide.

Pseudocode for the Zhang–Suen Thinning Algorithm:

function ZhangSuenThinning(image):

  changing1 = changing2 = true

  while changing1 or changing2:

    changing1 = []

    for each pixel (x, y) in the image:

      if image(x, y) is black:

        neighbors = count_neighbors(image, x, y)

        transitions = count_transitions(image, x, y)

        if 2 <= neighbors <= 6 and transitions == 1:

          if image(x, y + 1) * image(x − 1, y) * image(x, y − 1) == 0:

            if image(x − 1, y) * image(x + 1, y) * image(x, y − 1) == 0:

              changing1.append((x, y))

    for each (x, y) in changing1:

      image(x, y) = white

    changing2 = []

    for each pixel (x, y) in the image:

      if image(x, y) is black:

        neighbors = count_neighbors(image, x, y)

        transitions = count_transitions(image, x, y)

        if 2 <= neighbors <= 6 and transitions == 1:

          if image(x, y + 1) * image(x − 1, y) * image(x, y − 1) == 0:

            if image(x − 1, y) * image(x + 1, y) * image(x, y + 1) == 0:

              changing2.append((x, y))

    for each (x, y) in changing2:

      image(x, y) = white

function count_neighbors(image, x, y):

  return sum([image(x − 1, y), image(x − 1, y + 1), image(x, y + 1), image(x + 1, y + 1),

        image(x + 1, y), image(x + 1, y − 1), image(x, y − 1), image(x − 1, y − 1)])

function count_transitions(image, x, y):

  neighbors = [image(x − 1, y), image(x − 1, y + 1), image(x, y + 1), image(x + 1, y + 1),

        image(x + 1, y), image(x + 1, y − 1), image(x, y − 1), image(x − 1, y − 1)]

  transitions = 0

  for i in range(8):

    if neighbors[i] == white and neighbors[(i + 1) % 8] == black:

      transitions += 1

  return transitions

In the above pseudocode: function image(x, y) represents the pixel value at position (x, y) in the binary image (black for object pixel and white for background pixel). Function count_neighbors(image, x, y) returns the number of black neighbors for the pixel at (x, y). Function count_transitions(image, x, y) returns the number of transitions from white to black in the sequence of the eight neighbors around the pixel (x, y).

## Appendix B. Numerical Calculation Methods for the Roberts, Sobel, and Prewitt Operators

The Roberts operator is a simple and effective edge detection operator used to detect edges in digital images. It uses two 2 × 2 convolution kernels to convolve with the image to detect edges in the horizontal and vertical directions. It is mainly used to detect parts of the image with large intensity changes and is typically used for grayscale images.

The Roberts operator uses two convolution kernels:

1. $G_{x}=\left[\begin{array}{cc} +1 & 0\\ 0 & -1 \end{array}\right]$
2. $G_{y}=\left[\begin{array}{cc} 0 & +1\\ -1 & 0 \end{array}\right]$

These kernels calculate the gradient in the horizontal and vertical directions, respectively.

Pseudocode for the Roberts operator:

for each pixel (x, y) in the image:

  gx = image(x, y) − image(x + 1, y + 1)

  gy = image(x + 1, y) − image(x, y + 1)

  magnitude = sqrt(gx^2 + gy^2)

  if magnitude > threshold:

    output(x, y) = magnitude

  else:

    output(x, y) = 0

In the above pseudocode: function image(x, y) represents the pixel value at position (x, y) in the image. Symbols gx and gy are the gradients in the horizontal and vertical directions, respectively. Symbol magnitude is the magnitude of the gradient, representing the edge strength. Symbol threshold is a value used to control the sensitivity of edge detection. Only when the gradient magnitude exceeds the threshold is the point marked as an edge.

The Sobel operator is a commonly used edge detection operator that uses two 3 × 3 convolution kernels to improve the accuracy and stability of edge detection. The Sobel operator calculates the magnitude of the image gradient to detect edges and is typically used for grayscale images.

The Sobel operator uses two convolution kernels:

1. $G_{x}=\left[\begin{array}{ccc} -1 & 0 & +1\\ -2 & 0 & +2\\ -1 & 0 & +1 \end{array}\right]$
2. $G_{y}=\left[\begin{array}{ccc} -1 & -2 & -1\\ 0 & 0 & 0\\ +1 & +2 & +1 \end{array}\right]$

These kernels calculate the gradient in the horizontal and vertical directions, respectively.

Pseudocode for the Sobel operator:

for each pixel (x, y) in the image:

  gx = image(x − 1, y − 1)*(−1) + image(x − 1, y)*(0) + image(x − 1, y + 1)*(+1) +

     image(x, y − 1)*(−2) + image(x, y)*(0) + image(x, y + 1)*(+2) +

     image(x + 1, y − 1)*(−1) + image(x + 1, y)*(0) + image(x + 1, y + 1)*(+1)

  gy = image(x − 1, y − 1)*(−1) + image(x, y − 1)*(−2) + image(x + 1, y − 1)*(−1) +

     image(x − 1, y)*(0) + image(x, y)*(0) + image(x + 1, y)*(0) +

     image(x − 1, y + 1)*(+1) + image(x, y + 1)*(+2) + image(x + 1, y + 1)*(+1)

  magnitude = sqrt(gx^2 + gy^2)

  if magnitude > threshold:

    output(x, y) = magnitude

  else:

    output(x, y) = 0

In the above pseudocode: function image(x, y) represents the pixel value at position (x, y) in the image. Symbols gx and gy are the gradients in the horizontal and vertical directions, respectively. Symbol magnitude is the magnitude of the gradient, representing the edge strength. Symbol threshold is a value used to control the sensitivity of edge detection. Only when the gradient magnitude exceeds the threshold is the point marked as an edge.

The Prewitt operator is an edge detection algorithm based on edge strength. This algorithm first defines the kernels of horizontal and vertical edges. Then, it uses these kernels to convolve with the input image to calculate the horizontal and vertical edge strengths separately. Next, it calculates the total strength of the edges and applies a threshold to determine the presence of the edges. Finally, it outputs the detected edge image.

Prewiit_Edge_Detection(image : Matrix) : Matrix

  kx := [−1 0 1

     −2 0 2

     −1 0 1]

  ky := [−1 −2 −1

     0 0 0

     1 2 1]

  edge_x := create_empty_matrix(image.width, image.height)

  edge_y := create_empty_matrix(image.width, image.height)

  edge_magnitude := create_empty_matrix(image.width, image.height)

  for each pixel in image {

    edge_x[pixel] = convolve(image, kx, pixel)

    edge_y[pixel] = convolve(image, ky, pixel)

  }

  for each pixel in image {

    edge_magnitude[pixel] = sqrt(edge_x[pixel]^2 + edge_y[pixel]^2)

  }

  for each pixel in image {

    if edge_magnitude[pixel] > threshold {

      edge_magnitude[pixel] := white

    } else {

      edge_magnitude[pixel] := black

    }

  }

  return edge_magnitude

end function

## Appendix C. Pseudocode for RETC Algorithm

Initialize an empty list ‘radii’

For each skeleton point ‘center’, indexed by ‘i’:

  For radius from 1 to 9 (range can be adjusted):

    Initialize ‘intersected’ to False

    For each edge point ‘edge_point’, indexed by ‘j’:

      Calculate the distance ‘distance’ between the skeleton point and the edge point

      If ‘distance’ is less than or equal to the current radius:

        Set ‘intersected’ to True

        Break out of the edge points loop

    If an intersection is found (‘intersected’ is True):

      Add the current radius to the ‘radii’ list

      Print the skeleton point index ‘i’ and the radius

      Break out of the radius loop

Calculate the vessel diameter list ‘diameters = [2 * radius − 1 for radius in radii]’

If the ‘diameters’ list is not empty:

  Calculate and print the average diameter ‘average_diameter’

Else

  Print “No intersection found”
